# Integrative Nursing: Application of Principles Across Clinical Settings

**DOI:** 10.5041/RMMJ.10200

**Published:** 2015-04-29

**Authors:** Mary Jo Kreitzer

**Affiliations:** Director, Center for Spirituality & Healing; Professor, School of Nursing, University of Minnesota, Minneapolis, MN, USA

**Keywords:** Complementary therapies, integrative medicine, nurses, nursing, patient care

## Abstract

While the essence of nursing has long been whole person (body, mind, and spirit) and whole system-focused, in reality the contemporary practice of nursing in many settings around the globe has become increasingly fragmented and de-stabilized. Nursing shortages in many parts of the world are significant, and hierarchies and bureaucracies often remove nurses from the point of care, be that the bedside, home, or clinic, replacing them with less skilled workers and filling their time with documentation and other administrative tasks. Integrative nursing is a framework for providing whole person/whole system care that is relationship-based and person-centered and focuses on improving the health and wellbeing of caregivers as well as those they serve. It is aligned with what is being called the “triple aim” in the United States—an effort focused on improving the patient experience (quality and satisfaction), improving the health of populations, and reducing the cost of care. The principles of integrative nursing offer clear and specific guidance that can shape and impact patient care in all clinical settings.

## INTRODUCTION

From its earliest beginnings, nursing has been a holistic discipline focused on caring and healing. Florence Nightingale, often referred to as the founder of modern nursing, noted in the late 1800s that the role of the nurse was to put the patient in the best possible condition so that nature could act and healing occur.^1^ While the essence of nursing has long been whole person (body, mind, and spirit) and whole system-focused, in reality, the contemporary practice of nursing in many settings around the globe has become increasingly fragmented and destabilized. Nursing shortages in many parts of the world are significant,^2^ and hierarchies and bureaucracies often remove nurses from the point of care, be that the bedside, home, or clinic, replacing them with less skilled workers and filling their time with documentation and other administrative tasks. Technology, while life-saving, also has the potential of creating distance between the nurse and the patient, particularly when the nurse attends more to the “machine” than the patient.

Patients tell this story from a different point of view. Many experience a long parade of “care providers” who are too busy to actually care. Or, they encounter different nurses on every shift day after day, making it very difficult to establish a relationship that builds trust and confidence. In many settings, care has become so fragmented that care coordinators or care managers are required to maintain some semblance of order.

Stress in the health care environment also takes a toll on nurses. Burnout is a term used to describe workers’ reaction to chronic stress and is characterized by emotional exhaustion, depersonalization, and reduced personal accomplishment.^3^ Burnout among nurses has been reported to be higher than among other health professionals.^4^ As noted by Khamisa et al.,^5^ nursing requires the delivery of humane, empathetic, culturally sensitive, proficient, and moral care in working environments with limited resources and increasing responsibilities. When nurses experience burnout, it impacts their personal wellbeing as well as the quality and efficacy of patient care. Nurses experiencing ongoing stress are more likely to eat poorly, smoke cigarettes, and abuse alcohol and drugs.^6^,^7^ Lack of self-care is a pervasive issue that adversely impacts personal health and wellbeing, patient care, and the organization as a whole.

Integrative nursing is a framework for providing whole person/whole system care that is person-centered and relationship-based and focuses on improving the health and wellbeing of caregivers as well as those they serve. It is aligned with what is being called the “triple aim” in the United States—an effort focused on improving the patient experience (quality and satisfaction), improving the health of populations, and reducing the cost of care.^8^ The principles of integrative nursing offer clear and specific guidance that can shape and impact patient care in all clinical settings.^9^

## INTEGRATIVE NURSING

Within nursing, there are many broad theories based on concepts such as caring,^10^ unitary human beings,^11^ adaptation,^12^ self-care,^13^ human becoming,^14^ and health as expanding consciousness,^15^ to name a few. Theories aim to describe, predict, and explain the phenomenon of nursing. It is common for schools of nursing and departments of nursing services in clinical settings to adopt a theory and align a nursing practice model with the theory. While often the focus of student papers and the underlying framework for research studies, it is debatable how much nursing theory directly impacts care. Wadensten and Carlsson conducted a review of 17 well-known nursing theories originating over a 30-year period from the early 1960s to the early 1990s in order to delineate the views on aging and whether the theories provided insight or descriptions of how nursing care of older people could be organized.^16^ They concluded that while important aspects of nursing care of older people were discussed, no concrete instructions were given on how to apply the concepts to actual care.

While theory development has been very important in the development of nursing science, nurses in practice often view theory as being abstract and unrelated to their day-to-day practice. At a time when the need for nursing globally is so great and health care systems so fragmented and resources constrained, patients are demanding care that is more holistic and attentive to the whole person. Furthermore, nurses are yearning to provide care that is aligned with the values that led them into the profession originally. Integrative nursing is defined as “a way of being-knowing-doing that advances the health and wellbeing of persons, families and communities through caring and healing relationships.”^9^ Integrative nursing, a framework based on a set of principles that are consistent and aligned with major nursing theories, provides practical and unambiguous guidance that can both shape and direct the care of patient populations across clinical settings. While not intended in any way to replace nursing theory, the integrative nursing principles complement various theoretical paradigms and can be used concurrently.

## INTEGRATIVE NURSING PRINCIPLES AND BEHAVIORAL INDICATORS

The six principles of integrative nursing are based on meta-theoretical perspectives consistent with historical nursing values, beliefs, and theoretical perspectives; complex systems science; and the values, beliefs, and practices of integrative health care.^17^–^21^ The principles, above all, are practical. They address what nurses do and why they do it. The behavioral indicators illustrate concrete manifestations of the implementation of each principle. While many of the indicators below are applicable in virtually any setting, clinical settings are encouraged to develop specific indicators unique to their setting that can be measurable.

### 1. Human beings are whole systems inseparable from their environments

Simply stated, people are dynamic, individualistic, and complex and, as such, cannot be reduced to diagnoses, symptoms, and deviations from norms. Caring for the “whole person” requires attentiveness to the body, mind, and spirit and the interconnectedness of people to their environment. The environment encompasses all that surrounds the person, including the nurse, family, community, the built environment, and physical and metaphysical environments.

#### Examples of Nursing Care and Practice Indicators

The nurse:

- Completes a comprehensive assessment that includes all domains of health—physical, mental, emotional, and spiritual.
- Develops a plan of care that reflects the patient’s and family’s unique needs, strengths, and preferences to assure that care is coordinated and personalized.
- Promotes independence and offers choices.
- Maintains the integrity of the environment by monitoring noise, smells, and temperature and providing privacy.
- Personalizes the environment through art, use of personal objects, and accommodation of patient preferences.
- Becomes aware of attitudes, actions, and body language, recognizing that when caring for others the nurse becomes part of the environment of the patient.
- Uses mind/body approaches to become calm, centered, and fully present.
  ➢ As you wash your hands before entering the room, think about the patient you are about to see and feel a sense of gratitude for what your hands do as you care for patients.
  ➢ Take a few deep breaths before preparing medications for a patient.
  ➢ Pause and enjoy a view out of a window or appreciate a piece of art.
  ➢ Sit down during a break and feel the chair below you and your feet on the ground.

### 2. Human beings have the innate capacity for health and wellbeing

The body has healing and restorative capacities on many levels. When the integrity of the skin is damaged by a cut, scrape, or deeper wound, the body automatically goes into a process of inflammation, cell proliferation, and ultimately cellular repair.^22^ While neurons do not divide and are not capable of mitosis after injury, surviving nerve cells reorganize and establish new neural connections.^23^

Our mind has the capacity to help us heal. The brain has a property called neuroplasticity and is capable of changes in structure and function. Changes can occur as a result of experiences as well as purely internal mental activity, our thoughts.^24^ Positive emotions flood our brains with dopamine and serotonin, enhance immune system functioning, diminish the inflammatory response to stress, and change the scope and boundaries of the brain.^25^

People have the capacity to heal from deep psychological, emotional, and spiritual traumas and the accompanying grief, loss, anger, sadness, and despair. Kindness, compassion, caring, and love are human processes that can support healing when they are offered by others and when we care for ourselves in this way.

#### Examples of Nursing Care and Practice Indicators

The nurse:

- Nurtures the growth of hope, trust, and belief.
- Facilitates connections and relationships that lead to deeper meaning and wholeness.
- Engages and supports patient and family strengths.
- Cultivates presence.
- Focuses intention for healing and wholeness during ordinary nursing procedures.

### 3. Nature has healing and restorative properties that contribute to health and wellbeing

According to the biophilia hypothesis, human beings are innately drawn to nature and the natural world, and nature has properties that are healing and restorative.^26^,^27^ Recent systematic literature reviews support the growing evidence that being in nature is associated with reduction in blood pressure as well as reduced heart rate and respiratory distress/shortness of breath, with preliminary evidence pointing to changes in biological markers associated with the stress response and changes in neurological activity and brain activation.^28^,^29^ Recognizing the healing power of nature, evidence-based design of health care facilities is incorporating elements of biophilic design, including the use of natural light, color, art, and architectural features, such as curves, that mimic nature. There is significant growth in nature-based therapeutics, including the use of labyrinths, healing gardens, animal-assisted interactions, and facilitated green exercise.

#### Examples of Nursing Care and Practice Indicators

The nurse:

- Provides access to natural light.
- Opens a window for fresh air. Reduces exposure to environmental toxins, noxious stimuli (smells, noise, disturbances, allergens), providing a physical environment that is supportive and reduces harm.
- Integrates art and guided imagery that draws upon nature.
- Facilitates green exercise and spending time outdoors.
- Encourages the use of labyrinths and healing gardens and spaces.

### 4. Integrative nursing is person-centered and relationship-based

Caring and healing relationships are characterized by empathy, caring, love, warmth, trust, confidence, credibility, honesty, kindness, respect, and authentic communication. Person-centered care focuses on care of the whole person—body, mind, and spirit. Relationship-based care is built on continuity over time and calls upon the nurse to be fully present, to listen deeply, and to establish an authentic connection with the patient and family. When there is not continuity of care, i.e. a different nurse every shift over the course of a hospitalization or care encounter, it is exceedingly difficult if not impossible to provide person-centered and relationship-based care. Providing care of this nature requires a deep knowledge and connection with those we serve.

#### Examples of Nursing Care and Practice Indicators

The nurse:

- Knows the patient’s story and context.
- Greets patients and family members by name.
- Utilizes appropriate eye contact and touch.
- Anticipates and supports patients’ needs and preferences.
- Sets an intention to be a healing presence each shift, and comes back to that intention before interacting with each patient.
- Practices authentic listening: listening to learn, suspending judgment, and developing self-awareness.
- Connects as human beings, recognizing the patient as a person and the self as a person.
- Takes time during conversation for silence and reflection.
- Develops staffing and scheduling patterns that lead to continuity of nurse/patient relationships and care.
- Reflects on nurse–patient encounters to deepen the relationship.

### 5. Integrative nursing is informed by evidence and uses the full range of therapeutic modalities to support/augment the healing process, moving from least intensive and invasive to more, depending on need and context

Over the past 20 years, there has been a significant growth in the use of integrative therapies and healing practices.^30^–^33^ The drivers of this global phenomenon are many and include the limitations of Western medical approaches in managing symptoms, particularly of chronic disease, and the desire of people to use non-pharmacologic approaches to improve their health and wellbeing. Many of the so-called integrative therapies fall within the scope of nursing practice, and in the US some state nurse licensing boards have developed specific statements that acknowledge the use of integrative therapies.

#### Examples of Nursing Care and Practice Indicators

The nurse:

- Personalizes interventions based on the patients’ needs, wants, and preferences.
- Uses an evidence-informed approach to practice that supports selection of appropriate interventions or therapeutics, including the use of integrative therapies and healing practices.
- Uses integrative therapies and healing practices to manage symptoms and improve clinical outcomes and quality of life.
- Focuses nursing care on supporting the healing process.

In the book *Integrative Nursing*,^9^ a major focus is on the use of integrative therapies to manage symptoms more effectively. In discussing integrative symptom management, Ringdahl notes that a range of approaches and evidence-based healing modalities should be considered.^34^ Biomedical management of symptoms frequently begins with a pharmacological intervention intended to suppress symptoms and “fix” the problem. Integrative nursing shifts the focus from curing to healing, thus changing the nature of problem solving and prioritizing. This does not imply that biomedical interventions are discarded; rather they are introduced when that level of intervention is warranted. Nurses practicing from an integrative perspective are not merely “adding on” integrative therapies. Rather, integrative therapies become core to their practice.

Cutshall and Van Getson describe the evidence base underlying the use of integrative therapies for the management of nausea that includes dietary interventions, aromatherapy oils (ginger and peppermint), mind–body approaches (guided imagery, relaxation, hypnosis, and deep breathing), acupressure (P6 acupressure point), and energy healing such as Reiki.^35^ Non-pharmacologic pain management approaches include mind–body interventions such as the relaxation response, guided imagery, and mindfulness-based stress reduction as well as acupressure and acupuncture, yoga and movement therapies, massage, and access to nature.^36^ Integrative approaches are also described for the management of stress, sleep, anxiety, depressed mood, fatigue, cognitive impairment, and care of the human spirit.

### 6. Integrative nursing focuses on the health and wellbeing of caregivers as well as those they serve

As noted earlier, nurses work in intense, high-stress environments and are vulnerable to stress and burnout that impacts their own health and wellbeing as well as the care of patients. Self-awareness and self-care are core practices that are foundational to integrative nursing. Self-awareness allows nurses to notice inner experiences as they engage in caring for patients. Self-reflection, a form of ongoing inquiry, can lead to deeper learning and insights. Self-care is the most sustainable health care practice and comprises attentiveness to lifestyle behaviors (including healthy eating, exercise, sleep, and stress management) and may include the use of integrative therapies such as meditation, yoga, energy therapies, and massage.

#### Examples of Nursing Practice Indicators

The nurse:

- Develops and implements a personal plan for health and wellbeing.
- Engages in reflective practices such as journaling.
- Incorporates self-care practices into work and life situations.

## APPLICATION OF INTEGRATIVE NURSING ACROSS CLINICAL SETTINGS

The principles of integrative nursing have been readily implemented in a variety of clinical settings caring for diverse patient populations. A brief description of several sites illustrates the ease and guidance with which the principles can be applied.

### Woodwinds Health Campus

Woodwinds^37^ is an 86-bed community-based hospital that is part of the larger HealthEast system in St. Paul, Minnesota, USA. Adjacent to the hospital are primary and specialty clinics and the Natural Care Center, which offers chiropractic and nature-pathic medicine, massage, traditional Chinese medicine, and healing touch. Access to nature is evident in the architecture of the building and the placement of the campus within a large green space. The hospital incorporates many elements of biophilic design and provides access to nature-based artwork, healing gardens, and a labyrinth. Every room is private and has views of nature. The chapel embraces the visual support of earth, wind, fire, and water to embody the desire of a spiritually inclusive environment. Local Ojibwa elders assisted in the blessing of the healing space and healing gardens. Some departments have aquariums to invoke the water of the land, and multiple fireplaces provide warmth and light in most family and sitting areas, including the emergency waiting room. The overall design intentionally calls forth the iconic north woods cabins of Minnesota. Therapies offered within the healing arts program include healing touch, massage, guided imagery, use of essential oils, and healing music. Licensed acupuncture practitioners, certified massage therapists, and certified music practitioners are also available. The healing health care model has a strong focus on self-care and healing for the staff as a primary contribution to the overall healing environment.

### University of Minnesota Pediatric Blood and Marrow Transplant Center

The University of Minnesota Pediatric Blood and Marrow Transplantation Center^38^ cares for children around the world with life-threatening illnesses including leukemia, adrenoleukodystrophy, Hurler syndrome, Fanconi anemia, aplastic anemia, and epidermolysis bullosa. A decision was made in 2014 to incorporate integrative therapies into the team-based care from the time of diagnosis throughout the trajectory of care. An advanced practice nurse clinician with a Doctorate of Nursing Practice in integrative health and healing sees patients and their families when first diagnosed in the outpatient clinic and incorporates integrative therapies and healing practices into their overall plan of care. On the in-patient unit, nurses are educated to apply the principles of integrative nursing, as this whole-person framework is central to the practice model of the entire hospital.

### Touchstone Mental Health

Touchstone Mental Health provides person-centered care to individuals whose lives are affected by mental illness.^39^ Their mission is to inspire hope, healing, and wellbeing. The interdisciplinary team includes professionals from many disciplines including social work, nursing, psychiatry, psychology, counseling, chemical health, therapeutic recreation, nutrition, healing arts, and marriage and family therapy. Programs and services include adult rehabilitation mental health services, assisted living, care coordination, wellness and fitness centers, home and community-based services, intensive community rehabilitation services, intentional communities, residential treatment, and case management. The wellness center is led by an advanced practice nurse (with preparation as a nurse practitioner) who has expertise in integrative health and healing. Services within the wellness center include acupressure, acupuncture, massage, health coaching, yoga, and mind–body skills.

### The Waters Senior Living

The Waters Senior Living provides a full spectrum of care options that include independent living, assisted living, memory and Alzheimer’s care, and enhanced care.^40^ Still fairly unique in the rapidly changing industry of senior care is the focus on wellbeing and the use of integrative therapies and healing practices. According to the 2007 National Health Interview Survey,^41^ complementary and alternative medicine usage remained high among all subgroups of older adults: 41% of 60–69-year-olds, 32% of those aged 70–84, and 24% of those 85 and over. Older adults were most likely to use biologically based therapies, mind–body therapies, and manipulative and body-based therapies. In a study of community-dwelling older adults,^42^ the top four therapies used were nutritional supplements, spiritual healing or prayer, herbal supplements, and chiropractic care. The Waters employs Registered Nurses in all of the communities who practice integrative nursing.

## SUMMARY

Health care in many ways is at a cross-roads. The issues of rising costs, shortages of nurses and other health care providers, patients who are dissatisfied, poor outcomes, and disengaged care providers are global in nature. Addressing these issues requires a systems approach that will not be easy or fast. Integrative nursing provides a whole person/whole system approach that addresses the needs of patients and their families who are demanding care that is comprehensive, coordinated, and attentive to the whole person—body, mind, and spirit. Integrative nursing also engages nurses who yearn to practice in a way that is aligned with their personal values and the passion that ignited their call to a nursing career. It may be an effective strategy in buffering stress and attenuating the impact of burnout that is costly and takes a significant toll on patients, families, and caregivers. Health care today is a team-based endeavor, and nurses with the skills of integrative nursing are well positioned to be partners in delivering integrated health care.
